# Jasmonoyl-L-Isoleucine Induces Systemic Photosynthetic Responses to Local Heat Stress by Contributing to Abscisic Acid Accumulation

**DOI:** 10.3390/plants15111732

**Published:** 2026-06-03

**Authors:** Darya Kuznetsova, Vladimir Vodeneev, Maria Ladeynova

**Affiliations:** Department of Biophysics, National Research Lobachevsky State University of Nizhny Novgorod, 23 Gagarin Avenue, 603022 Nizhny Novgorod, Russia

**Keywords:** phytohormones, jasmonates, abscisic acid, photosynthesis, stomatal conductance, systemic response

## Abstract

Systemic regulation of photosynthesis is crucial for plant survival in variable environments, yet the hormonal mechanisms coordinating photosynthetic responses to local stimuli are not fully elucidated. This study investigates the interplay between jasmonates (JAs) and abscisic acid (ABA) in systemic photosynthetic responses induced by local heat stress in *Arabidopsis thaliana*. Using phytohormone quantification, chlorophyll fluorescence and gas exchange measurements in wild-type and transgenic plants impaired in JA biosynthesis, this study showed that local heating-induced variation potential propagation triggers JA biosynthesis in systemic unstimulated leaves, followed by changes in ABA content, stomatal conductance and photosynthetic activity. Rapid systemic increases in jasmonoyl-L-isoleucine (JA-Ile) levels are essential for the systemic decreases in stomatal conductance and the consequent reduction in carbon assimilation. Systemic increases in JA-Ile levels also contribute to systemic accumulation of ABA, likely to maintain reduced stomatal conductance. Thus, the data support a model in which JA-Ile acts as a mediator of early stages of the systemic photosynthetic response, whereas ABA likely contributes to late stages of this response. These results highlight the complex integration of hormonal signals in the regulation of photosynthesis under stress conditions.

## 1. Introduction

Rapid coordination of stress responses is essential for plant survival in a constantly changing environment. One of the most important plant stress responses is changes in photosynthetic activity [1,2]. Although there are still many gaps, substantial progress has been made in understanding the mechanisms that coordinate photosynthetic processes under abiotic stresses, including salinity, drought, and extreme temperatures [2,3,4]. However, highly complex photosynthetic regulation is involved in systemic responses to local stimuli such as exposure to excess light, heat stress, mechanical damage, and herbivore attack. In addition to photosynthetic responses in the site of local stimulation, photosynthetic activity also changes in distal unstimulated tissues [5,6,7]. Hence, the mechanisms leading to changes in photosynthetic activity include both direct and indirect effects.

Photosynthetic responses have been shown to differ between stimulated local and unstimulated systemic tissues because different signaling mechanisms are involved in the induction of these responses [7,8,9]. Local signaling involves damage-associated molecular patterns (DAMPs), whereas systemic signaling is based on long-distance signals such as waves of reactive oxygen species (ROS) and Ca^2+^, as well as hydraulic and electrical signals [10,11,12]. However, the mechanisms linking signaling events and photosynthetic responses in local and systemic tissues remain largely unclear.

There is evidence that short-term systemic photosynthetic responses are coordinated through the interplay between electrical signals, ROS, Ca^2+^ waves, and phytohormones [13,14,15,16]. Electrical and Ca^2+^ signaling are essential for the systemic biosynthesis of jasmonates (JAs), key phytohormones involved in defense responses to mechanical damage and herbivory [17,18,19,20,21]. One type of long-distance signals, the variation potential (VP; also termed slow wave potential), is a complex electrical signal induced by hydraulic and chemical signals [21,22]. VP propagation is associated with changes in pH and concentrations of signaling messengers such as Ca^2+^ and ROS [19,21,22,23]. VP is closely linked to the synthesis of JAs through alterations in ion concentrations that accompany its propagation [18,23,24]. Rapid long-distance signal transmission is required to initiate the biosynthesis of jasmonic acid (JA) and its bioactive form, (+)-7-*iso*-jasmonoyl-L-isoleucine (JA-Ile), in systemic tissues within minutes or tens of minutes after local stimulation [25,26,27,28]. However, the mechanisms initiating the production of other hormones, the content of which changes upon local stimulation and, thereby, can influence photosynthetic activity, remain largely unknown [15]. Along with JA, an important role of abscisic acid (ABA), salicylic acid (SA), 12-oxo-phytodienoic acid (OPDA), and cytokinins in photosynthetic regulation and systemic stomatal responses upon local stimulation has been demonstrated [7,8,13,29,30].

Stomatal closure is considered to be the main mechanism for the induction of photosynthetic responses by hormones [8,15]. Phytohormones can also influence photosynthesis by regulating Calvin cycle enzyme activity and glucose transport [31,32,33]. Furthermore, one of the possible mechanisms of regulation of photosynthesis by hormones is the control of CO_2_ diffusion by altering mesophyll conductance, as has been shown, in particular, for ABA [34,35]. The mechanisms of stomatal closure involving various hormones are different, but despite this, there are universal features of these mechanisms. These features include the key role of ROS and alterations in ion concentrations, primarily Ca^2+^. These signaling events result in a decrease in guard cell turgor due to a reduction in osmotic pressure, which is mainly mediated by the efflux of K^+^, Cl^−^ and malate ions from the guard cells [15,30,36].

Recent studies highlight that phytohormone action largely depends on the interplay of hormones and the integration of their signaling pathways, rather than on the individual activity of hormones [29,30,36,37,38]. It has been shown that photosynthetic responses induced by local stimuli can be mediated by complex interactions among JAs, ABA, and cytokinins [8,13]. However, the complexity of hormonal crosstalk, as well as contradictory data on the phytohormone dynamics upon local stimulation [28,39,40,41,42,43,44], indicates the need for further investigations to fully elucidate the regulatory mechanisms of systemic photosynthetic responses.

This work examines the role of phytohormone crosstalk in the regulation of systemic photosynthetic responses induced by local heat stress. By combining phytohormone quantification with knowledge of the vascular architecture of *Arabidopsis thaliana*, this study reveals the spatial and temporal coherence of JA and ABA dynamics that depend on the pattern of vascular connections between leaves. Using molecular approaches with transgenic lines of *Arabidopsis* plants with impaired biosynthesis of JAs, the involvement of JAs in the systemic ABA production in response to local stimulation was identified. The analysis of systemic photosynthetic responses induced by local heating revealed the interaction of ABA and JAs to modulate changes in photosynthetic gas exchange.

## 2. Results

### 2.1. Spatial and Temporal Changes in Phytohormone Content upon Local Heating

**Figure 1:**
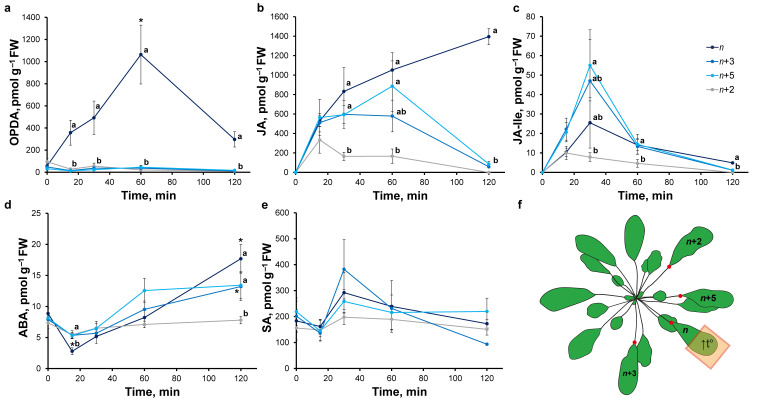
Spatiotemporal dynamics of 12-oxophytodienoic acid (OPDA) (**a**), jasmonic acid (JA) (**b**), jasmonoyl-isoleucine (JA-Ile) (**c**), abscisic acid (ABA) (**d**) and salicylic acid (SA) (**e**) induced by heating of the leaf *n* in wild-type *Arabidopsis* plants (Col-6). The variation potential (VP) propagation was monitored simultaneously with the analysis of hormones. The moment of VP propagation corresponds to the time point “0 min”. The same plants were used as a single experimental set for VP recording and phytohormone sampling. Data are represented as Mean ± SEM (*n* = 3 to 6). Asterisks indicate data significantly different from unstimulated plants (0 min) according to one-way ANOVA analysis followed by Dunnett’s test (*p* < 0.05). Different letters indicate statistically significant differences between leaves within a time point according to Student’s *t* test (*p* < 0.05). (**f**) Experimental design for detecting changes in electrical potentials in leaves of *Arabidopsis* plants. Red circles indicate surface electrodes. The apical part of leaf *n* was heated.

The levels of ABA, SA, and JAs were quantified at different time points after local heating in stimulated (local) and unstimulated (systemic) leaves of wild-type (WT) *Arabidopsis* plants, namely the Col-6 (Figure 1) and the Col-0 (Figure S1). Since long-distance signals are essential to trigger responses in systemic tissues, monitoring of electrical potentials was used to control VP propagation in the studied leaves. Heating of the apical part of leaf *n* induced generation of VP in this leaf, which then propagated to distal leaves *n* + 5, *n* + 3 and *n* + 2 (Figure S2). In the Col-6 line, changes in the content of ABA (Figure 1d), JA and JA-Ile (Figure 1b,c) were found in both local and systemic tissues, but significant changes in OPDA levels were observed only in the local leaf (Figure 1a). No significant differences were observed for SA either in local or in systemic leaves in response to local heating (Figure 1e).

OPDA levels in stimulated leaf *n* increased within 60 min after VP propagation and then decreased to resting levels at 120 min (Figure 1a). Peak levels of OPDA were 19-fold higher than pre-stimulation levels. JA and JA-Ile levels began increasing within 15 min after VP in both local and systemic leaves (Figure 1b,c), reaching a maximum 15–60 min after VP, except for the continuous increase in JA levels in local leaf *n*. The duration of stimulus-induced accumulation of JA was longer than that of JA-Ile. The highest increase in JA levels was in the stimulated leaf *n*. The amplitude of JA burst decreased from local leaf *n* to systemic leaf *n* + 2. The highest amplitude of JA-Ile burst was in leaves *n* + 3 and *n* + 5, and smaller in leaves *n* and *n* + 2. The lowest accumulation of JA and JA-Ile was observed in distal leaf *n* + 2.

ABA levels fell within 15 min after VP propagation and then gradually increased in the time interval from 15 to 120 min in local leaf *n* and systemic leaves *n* + 3 and *n* + 5, but not in systemic leaf *n* + 2 (Figure 1d). The highest amplitudes of decrease in ABA levels at 15 min and increase in ABA levels at 120 min were observed in the stimulated leaf *n*.

In the Col-0 line, similar spatiotemporal dynamics of phytohormones in response to local heating were found (Figure S1). Substantial changes in OPDA levels were observed only in the stimulated leaf, whereas changes in the concentrations of ABA, JA and JA-Ile were found in both local and systemic leaves, and SA levels were not significantly altered upon local heating. Similar to the Col-6 line, the Col-0 line exhibited rapid increases in JA and JA-Ile levels and later increases in ABA levels, the amplitudes of which were higher in the stimulated leaf *n* and systemic leaf *n* + 5 compared to the amplitudes in systemic leaf *n* + 2.

Analysis of the spatial dynamics of JA, JA-Ile and ABA in both the Col-6 and Col-0 lines revealed spatial heterogeneity of JA, JA-Ile and ABA content changes triggered by local heating. This heterogeneity followed a consistent pattern, with the maximum amplitude in leaf *n*, lower amplitudes in leaves *n* + 3 and *n* + 5, and the lowest amplitude or no change in leaf *n* + 2. This observation, together with the fact that JA and JA-Ile bursts preceded the increase in ABA levels (Figure 1b–d), suggests the involvement of JAs in the control of systemic ABA production upon local heating.

### 2.2. The Role of Jasmonates in the Regulation of Hormone Levels upon Local Heating

**Figure 2:**
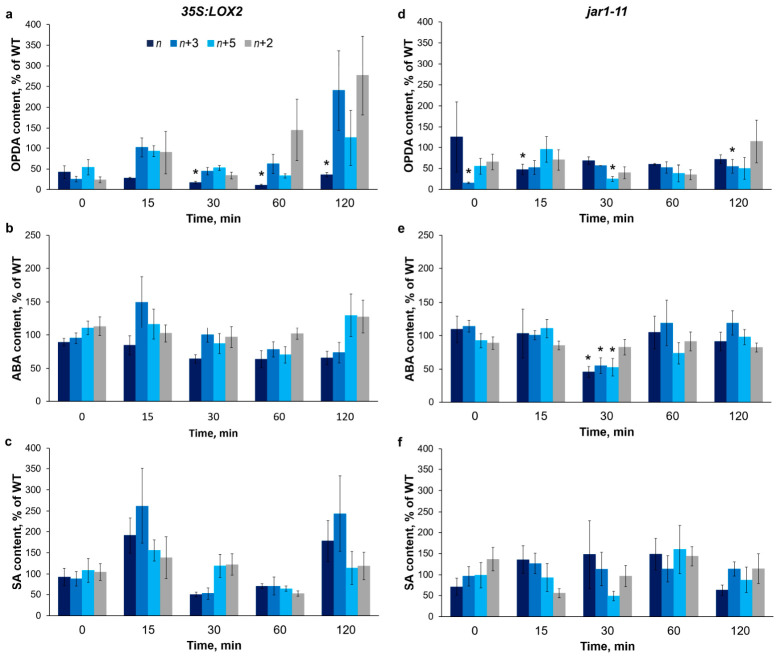
Spatiotemporal analysis of 12-oxo-phytodienoic acid (OPDA) (**a**,**d**), abscisic acid (ABA) (**b**,**e**) and salicylic acid (SA) (**c**,**f**) levels in *35S:LOX2* transgenic line (**a**–**c**) and *jar1-11* mutant (**d**–**f**) upon local heating. Data are represented as Mean ± SEM (*n* = 3 to 6). OPDA, ABA and SA levels in *35S:LOX2* and *jar1-11* are represented as the percentage of their respective levels in WT plants, which are the OPDA, ABA and SA levels in the corresponding background lines, namely Col-6 for *35S:LOX2* and Col-0 for *jar1-11*. Asterisks indicate data significantly different from WT within a time point according to Student’s *t* test (*p* < 0.05).

To elucidate the role of JAs in the regulation of stimulus-induced changes in ABA content, two transgenic lines of *Arabidopsis* plants impaired in the JA biosynthesis upstream and downstream of OPDA were used. The *35S:LOX2* line [45] was impaired upstream of OPDA, namely in the function of 13-lipoxygenase 2 (LOX2). In the *35S:LOX2* line, the *LOX2* gene is placed under the control of the 35S promoter to reduce *LOX2* expression. *35S:LOX2* transgenic plants have decreased *LOX2* mRNA and protein levels, and consequently reduced accumulation of JA, compared to corresponding background lines [45]. LOX2 is known to be required for the biosynthesis of a large pool of JAs in wounded *Arabidopsis* leaves [25,45]. *Jar1-11* mutant plants [46] were impaired downstream of OPDA, namely in the function of JASMONATE RESISTANT 1 (JAR1), which catalyzes the synthesis of the bioactive JA-Ile [47,48]. To evaluate the impact of LOX2 and JAR1 on systemic changes in hormone levels triggered by local heating, the hormone levels in *35S:LOX2* and *jar1-11* plants were represented as the percentage of WT levels. WT levels were determined in the corresponding background lines, namely Col-6 for *35S:LOX2* and Col-0 for *jar1-11* (Figure 2).

Quantification of OPDA in *35S:LOX2* plants under control conditions (without stimulation) using pooled leaves *n*, *n* + 2, *n* + 3, *n* + 5 revealed a reduction in OPDA resting levels by 67% from the WT (16.8 ± 4.0 pmol g^−1^ FW in WT, 5.5 ± 0.9 pmol g^−1^ FW in *35S:LOX*, *p* = 0.02), indicating the involvement of LOX2 in the formation of OPDA pools in *Arabidopsis* plants. Resting levels of OPDA in *jar1-11* plants remained unchanged compared to the WT. Similar to WT plants, local heating triggered a substantial increase in the content of OPDA in the stimulated leaf *n* of *35S:LOX2* and *jar1-11* plants (Figures S3a and S4a). However, the heating-induced OPDA levels in leaf *n* were strongly reduced to 10–36% of the WT levels in *35S:LOX2* plants but not in *jar1-11* plants (Figure 2a,d). Similar to WT, OPDA content in systemic leaves of *35S:LOX2* and *jar1-11* plants remained unchanged after local stimulation (Figures S3a and S4a). Compared with WT, OPDA levels in systemic leaves were not significantly altered in either *35S:LOX2* or *jar1-11* plants (Figure 2a,d), except in leaf *n* + 5 at 30 min and leaf *n* + 3 at 120 min after VP propagation in *jar1-11* plants. These findings indicate that LOX2 activity is required only for local production of OPDA, and JAR1 activity is likely not required for either local or systemic production of OPDA upon local heating.

Quantification of ABA in *35S:LOX2* and *jar1-11* plants showed the lack of changes in resting levels of ABA in both transgenic lines (Figure 2b,e), indicating that LOX2 or JAR1 activity is not required to produce basal ABA levels. Similar to WT plants, local heating induced an increase in the content of ABA in the stimulated leaf *n* and systemic leaves of *35S:LOX2* and *jar1-11* plants (Figures S3b and S4b). The spatial and temporal dynamics of ABA levels in leaves *n*, *n* + 2, *n* + 3, *n* + 5 of both transgenic lines were similar to those in WT plants (Figures S3b and S4b). Upon local heating, no statistically significant differences in ABA levels were observed between *35S:LOX2* and WT plants (Figure 2b), but there was a tendency toward reduced ABA levels in the local leaf *n* at 30, 60 and 120 min (Figure 2b), consistent with reduced OPDA levels in this leaf at the same time points (Figure 2a). In *jar1-11* plants, ABA levels were reduced relative to the WT 30 min after VP propagation in local leaf *n* and systemic leaves *n* + 3 and *n* + 5, but not in leaf *n* + 2 (Figure 2e). Moreover, at the same time point, a strong attenuation of heating-induced increases in JA-Ile levels was observed in local leaf *n* and systemic leaves *n* + 3 and *n* + 5, but not in the leaf *n* + 2 of *jar1-11* plants, as reported previously [49]. This is consistent with the peak of JA-Ile accumulation at 30 min (Figure 1c). Taken together, these findings indicate that JAs may act upstream of ABA to regulate its systemic production upon local heating.

SA content in *35S:LOX2* and *jar1-11* plants was not altered compared to WT plants in response to local heating (Figure 2c,f). Quantification of JA and JA-Ile was performed simultaneously with OPDA, ABA and SA. The obtained data on JA and JA-Ile levels were similar to those of the previous study [49].

### 2.3. Systemic Photosynthetic Suppression Mediated by Jasmonoyl-Isoleucine-Induced Reduction in Stomatal Conductance upon Local Heating

The aforementioned results demonstrated the strongest increases in the levels of JAs and ABA in local leaf *n* and systemic leaf *n* + 5 (Figure 1a–d). In leaf n, these changes and following responses are likely driven primarily by the direct effect of the stimulus [11], whereas in leaf *n* + 5, these changes appear to rely on long-distance signaling, which plays a pivotal role in systemic stress responses. Thus, particular focus was placed on systemic leaf *n* + 5 to identify the hormone-related mechanisms leading to changes in photosynthesis after local heating.

**Figure 3:**
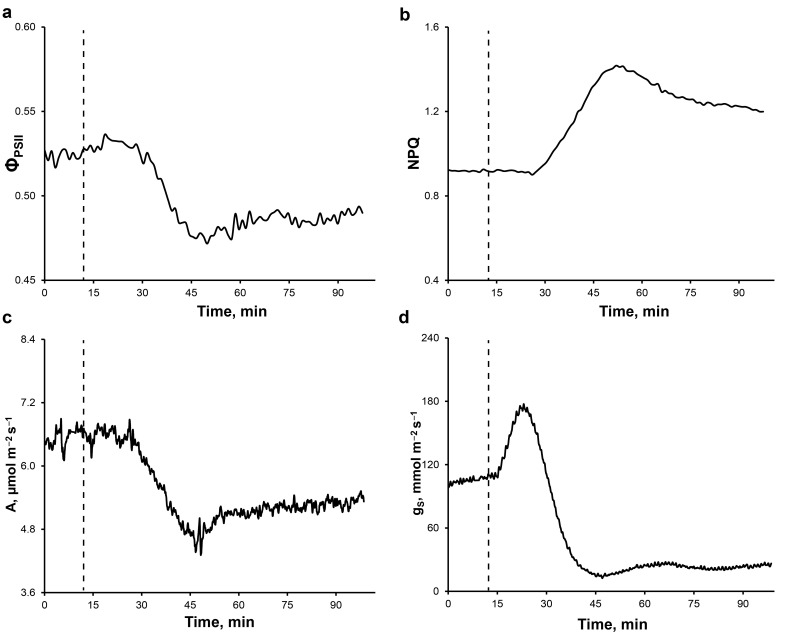
Representative recordings of systemic changes in chlorophyll fluorescence parameters (**a**,**b**), CO_2_ assimilation (**c**) and stomatal conductance (**d**) in response to local heating in wild-type *Arabidopsis* plants (Col-0). The dashed line indicates the moment of propagation of the variation potential. Φ_PSII_, effective quantum yield of photochemical reactions of photosystem II; NPQ, non-photochemical quenching of fluorescence; A, CO_2_ assimilation; g_S_, stomatal conductance.

To investigate the relationship between changes in photosynthetic activity and changes in stomatal conductance (g_S_), continuous and simultaneous monitoring of chlorophyll fluorescence and gas exchange parameters with high-temporal resolution was carried out. Heating of the local leaf *n* caused the changes in effective quantum yield of photochemical reactions of photosystem II (Φ_PSII_), non-photochemical quenching of fluorescence (NPQ), CO_2_ assimilation (A), and g_S_ in the systemic leaf *n* + 5 of WT *Arabidopsis* plants (Col-0) (Figure 3). Local heating induced suppression of photosynthetic activity, indicated by a decrease in A and Φ_PSII_, and an increase in NPQ. The decrease in Φ_PSII_ was biphasic and attained peak levels ~35 min and ~65 min after VP propagation, respectively (Figure 3a).

Changes in g_S_ began immediately after VP propagation and were multiphasic (Figure 3d), consisting of a short-term transient increase in g_S_ (activation phase) that lasted 15 min and followed by a long-term biphasic decrease (suppression phase). The decrease in g_S_ reached its maximum ~35 min after VP propagation in the first phase and ~65 min after VP in the second phase. The peak of the first phase of g_S_ suppression (Figure 3d) corresponds to the maximum decrease in Φ_PSII_ and A (Figure 3a,c), as well as with the maximum increase in NPQ (Figure 3b). Moreover, during the second suppression phase, the peaks of Φ_PSII_ and g_S_ also coincided (Figure 3a,d). Overall, these results suggest that systemic photosynthetic suppression was closely associated with, and likely substantially mediated by, a decrease in g_S_.

**Figure 4:**
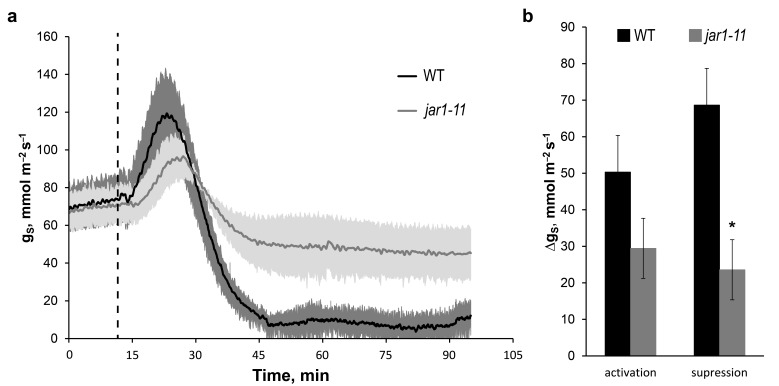
Averaged systemic responses of stomatal conductance (g_S_) induced by local heating (**a**) and amplitudes of g_S_ changes (**b**) in *jar1-11* mutant and wild-type *Arabidopsis* plants (Col-0 background line). The dashed line indicates the moment of propagation of the variation potential. Data are represented Mean ± SEM (*n* = 3). Asterisk indicates data significantly different from WT according to Student’s *t* test (*p* < 0.05).

Due to the key role of g_S_ in mediating stimulus-induced photosynthetic responses, systemic changes in g_S_ were monitored in the *jar1-11* transgenic line with impaired biosynthesis of JA-Ile, and compared with the corresponding Col-0 background line (Figure 4). Under control conditions (without stimulation), g_S_ in *jar1-11* plants was statistically indistinguishable from that in WT plants. Similar to WT *Arabidopsis* plants, local heating induced multiphasic changes in g_S_ in the systemic leaf *n* + 5 of *jar1-11* plants (Figure 4a). However, *jar1-11* plants exhibited an impaired systemic g_S_ response to local heating, with a 63% reduction in the amplitude of the stimulus-induced decrease in g_S_ (Figure 4b), consistent with the previous results [44] obtained using thermal imaging. The amplitude of the stimulus-induced initial increase in g_S_ remained unchanged in *jar1-11* plants compared to the WT (Figure 4b). These findings indicate that the systemic decrease in g_S_ upon local heating is induced by JA-Ile whose formation is mediated by JAR1 activity.

## 3. Discussion

The results of this study demonstrated systemic changes in phytohormone content in response to local stimulation, which has also been documented in many other studies [7,15,42,43,50,51,52]. The dependence of stimulus-induced changes in JA and JA-Ile levels on a specific pattern across leaves of *Arabidopsis* plants (Figure 1b,c) has been well described in many works [17,25,41,42,50,53,54] and can be explained by interleaf vascular connections, termed as parastichies [55]. The most pronounced increases in systemic JA and JA-Ile levels were observed in leaf *n* + 5 (Figure 1b,c), which shares direct vascular connections with the stimulated leaf *n* [55]. In contrast, leaf *n* + 2, which is a less directly connected leaf [55], showed the smallest increase (Figure 1b,c). This pattern of JA and JA-Ile changes is controlled by long-distance signals, most likely VP, whose amplitude and duration depend on the vascular pathways, since VP propagates through the vasculature [18,19,21,23].

In contrast to JA and JA-Ile, an increase in OPDA levels was observed only in the stimulated leaf *n* (Figure 1a–c). This study showed a transient increase in local OPDA levels (Figure 1a), consistent with observations from another work using *Nicotiana benthamiana* and local mechanical wounding as a research model [44]. However, in other studies, mechanical wounding triggered a continuous increase [26,56] or a rapid decline in OPDA content [51] in *Arabidopsis* plants. Here, investigations of spatiotemporal dynamics did not reveal changes in OPDA levels in systemic leaves (Figure 1a), which is consistent with other published data on mechanical wounding [41], heat wounding [42] and herbivore wounding [57]. However, a rapid decline in OPDA content in *Arabidopsis* plants upon wounding [26,51] or a short-term increase, followed by a rapid decrease in wheat plants upon local heating [24] have also been reported regarding systemic tissues. The lack of statistically significant differences in systemic OPDA levels between stimulated and unstimulated plants (Figure 1a) does not allow us to suggest a possible role of VP in inducing changes in OPDA levels after local stimulation. Nevertheless, the previous study suggested that depolarization during VP triggers systemic decreases in OPDA levels [57], as evidenced by analysis of OPDA content in mutants deficient in *GLUTAMATE RECEPTOR-LIKE* (*GLR*) genes [57], which cannot propagate VPs to leaves distal to damage sites [17].

Although extensive research has been conducted on JAs, less is known about the dynamics of other phytohormones upon local stimulations. SA levels were not altered in either local or systemic leaves (Figure 1e). Therefore, SA is unlikely to be involved as a hormone mediating short-term photosynthetic responses to local heating both locally and systemically. The lack of changes in SA content (Figure 1e) is consistent with data on the mechanical wounding of *Arabidopsis* plants [41] and local burning of *Nicotiana tabacum* [39]. Nevertheless, there is evidence of increased SA content in local and systemic tissues in response to local stimulation [15].

Similar to JA and JA-Ile, the stimulus-induced ABA distribution pattern is linked to the vascular connections between *Arabidopsis* leaves (Figure 1d). This observation is consistent with previous results showing that local burning of a pea leaf induced VP propagation followed by a rapid JA burst and late ABA accumulation in systemic leaves [58]. At the same time, the amplitudes of VP, as well as increases in JA and ABA levels, depended on the distances of vascular connections between the pea leaves [58]. This suggests that changes in ABA levels are either controlled by long-distance signals, or regulated by JA/JA-Ile, or both. To date, the mechanisms of induction of systemic changes in ABA levels upon local stimuli remain far from clear. Moreover, available data on ABA dynamics is limited to a small number of works and rather contradictory. Overall, local stimuli induced an increase in ABA content in both local and systemic tissues [15]. In *Arabidopsis*, heat wounding caused a higher increase in ABA levels in the local leaf than in systemic leaves [42]. This pattern is also reproduced in the present work (Figure 1d). However, a significant decrease in ABA levels was also observed in the systemic leaf *n* + 5 of *Arabidopsis* plants after mechanical wounding [41]. Furthermore, wounding induced a decrease in ABA content followed by an increase in ABA content in tubers of *Solanum tuberosum* [59]. In this work, a rapid stimulus-induced decrease in the ABA level in local leaf was observed (Figure 1d), which is likely due to direct heat exposure and/or changes in electrical activity. It has been proposed that electrical signals may influence ABA content through changes in Ca^2+^ concentrations and pH accompanying the generation of electrical signals [15]. In particular, pH changes can rapidly influence ABA content through conjugation and deconjugation of ABA glucosyl ester (ABA-GE) [15]. In turn, the late increases in ABA content (Figure 1d) are more likely to be induced by JAs rather than by rapid long-distance signals.

To determine the role of JAs in the induction of stimulus-induced increases in ABA content, ABA dynamics in local and systemic leaves of transgenic lines of *Arabidopsis* plants impaired in the JA biosynthesis and WT plants after local heating were compared quantitatively (Figure 2). It was found that *jar1-11* plants, which exhibited strongly reduced amplitudes of stimulus-induced JA-Ile increases as demonstrated in our previous study using a similar experimental design [49], also had altered ABA content upon local heating as shown in this study (Figure 2e). In *jar1-11* mutants, ABA levels were reduced relative to the WT 30 min after VP propagation (Figure 2e), coinciding with the peak increase in JA-Ile levels (Figure 1c), which was reduced in *jar1-11* plants compared to the WT, as shown in our previous study [49]. Subsequent increases in ABA levels (Figure 1d), corresponded to decreases in JA-Ile levels (Figure 1c) and were not affected in *jar1-11* mutants (Figure 2e), suggesting a potential role of JA-Ile in initiating ABA production upon local heating. Consistent with these findings, recent work has shown that ABA accumulates in wounded leaves of *Arabidopsis*, whereas disruption of JA signaling results in a reduction in ABA content after wounding compared to WT [60].

The pathway by which JA-Ile regulates ABA accumulation is currently not clear, but can be hypothesized based on the available published evidence discussed below. One of the pathways of JA-Ile-mediated induction of ABA production upon local stimuli is the regulation of ABA biosynthetic genes. It was shown that upon cold stress, in tomato, JA activates *MYC2*, a core component of the JA signaling pathway. In turn, *MYC2* promotes transcription of the ABA biosynthesis gene *9-CIS-EPOXYCAROTENOID DIOXYGENASE 2* (*NCED2*), which leads to ABA accumulation [61]. Furthermore, in mechanically wounded leaves of *Arabidopsis*, *NCED3* was activated by JA via *MYC2* [60]. More recent work also showed that JA and ABA signaling pathways converge in the stress response of detached *Arabidopsis* leaves, which are subjected to wounding and osmotic stress as a result of detachment [62]. These stress conditions upregulate *MYC2* and ABA signaling pathway transcription factor gene *ABA INSENSITIVE 5* (*ABI5*), which form the MYC2-ABI5 transcription factor complex that promotes the expression of *β-GLUCOSIDASE 18* (*BGLU18*), which releases ABA from ABA-GE, resulting in ABA signal amplification [62]. However, the involvement of these pathways in the interaction between JA and ABA in systemic tissues upon local stimulation is unknown and requires further research.

This study also showed that the impairment of JA biosynthesis in transgenic lines of *Arabidopsis* plants also affected the content of OPDA (Figure 2a,d), which is known not only as a JA precursor but also as a signaling molecule with functions independent of JA [63,64]. Heating-induced OPDA levels in the stimulated leaf of *35S:LOX2* plants were found to be significantly reduced compared to those in WT plants (Figure 2a). This finding is consistent with studies using the *lox2-1* mutant and mechanical wounding [25,65]. These studies showed that LOX2 was necessary for production of JAs in wounded leaves [25,27,65].

The impairment of JA biosynthesis in transgenic plants may also affect SA content [65] due to the well-known antagonistic relationship between SA and JAs [66,67]. However, contrary to expectations, plants defective in JA biosynthesis exhibited no significant differences in the SA content compared to WT plants (Figure 2c,f), which aligns with the absence of stimulus-induced changes in SA levels (Figure 1e and Figure S1e).

Taken together, these findings indicate that JA-Ile and ABA, but not SA, may play roles in systemic responses induced by local heating. In addition, OPDA, together with JA-Ile and ABA, may be involved in local photosynthetic responses, as suggested by the data reported here (Figure 1a and Figure 2a and Figure S3a) and published evidence [8,68].

Unlike OPDA, JA-Ile and ABA can mediate systemic photosynthetic responses to local stimuli. In turn, systemic photosynthetic responses induced by local heating were substantially mediated by a decrease in g_S_ (Figure 3), which was also identified in other works [7,8,52]. This study demonstrated multiphasic changes in g_S_ in response to local heating in *Arabidopsis* plants (Figure 3d), which were also observed in response to local heating and other local stimuli in wheat [6,69] and *Arabidopsis* [70]. The multiphasic nature of systemic changes in g_S_ suggests that individual phases of the response are induced by different signals, including different hormonal signals. The initial phase of activation of g_S_ may be mediated by hydropassive opening of stomata [43,71], whereas the suppression phase of g_S_ is more likely mediated by hormonal regulation.

The JA-Ile-induced systemic decrease in g_S_ in response to local heating has been previously reported [49]. However, in the present study, a biphasic decrease in g_S_ was observed (Figure 3d), suggesting the involvement of different hormone-related mechanisms, which operate at different temporal scales. The first wave of g_S_ decrease peaked ~35 min after VP propagation (Figure 3d), corresponding to the peak of JA-Ile accumulation (Figure 1c), and the second wave of g_S_ decrease peaked ~65 min after VP (Figure 3d), coinciding with the onset of ABA level increases (Figure 1d). It is also noteworthy that in WT plants, g_S_ remained persistently low for more than 60 min after VP (Figure 3d), whereas the stimulus-induced systemic increases in content of JA-Ile had already declined to low levels after more than 60 min (Figure 1c), but ABA content remained elevated in leaf *n* + 5 or increased in leaf *n* (Figure 1d). Thus, ABA may contribute to a further decrease in g_S_, which is consistent with the previously reported prolonged suppression of photosynthesis [49]. It is also well known that ABA promotes stomatal closure [71,72]. Collectively this suggests that ABA, as a long-term signal, may mediate systemic photosynthetic responses through a reduction in g_S_.

Investigations of stimulus-induced systemic changes in g_S_ in *jar1-11* mutants revealed that these changes are induced by JA-Ile. Remarkably, systemic g_S_ responses triggered by local heating were not completely abolished in *jar1-11* plants (Figure 4). At the same time, our previous study reported that systemic increases in JA-Ile levels caused by local heating were also not completely abolished in *jar1-11* plants and remained at 10–20% of the WT levels [49], which are likely sufficient to induce systemic g_S_ responses. An alternative possibility is that another signal induces systemic g_S_ responses in *jar1-11* plants. ABA, SA and cytokinins have been shown to be necessary for the regulation of JA-mediated changes in g_S_ and photosynthetic activity [8,13]. However, taking into account the substantial attenuation of g_S_ responses in *jar1-11* mutants (Figure 4b), it can be suggested that the main contribution is made by JA-Ile. The key role of JA-Ile in suppressing g_S_ upon local stimulation is supported by published data on JA signaling mutants other than *jar1* [7,29,73,74].

Systemic changes in g_S_ induced by JA-Ile are likely mediated by the JA receptor CORONATINE INSENSITIVE 1 (COI1) [29]. Local wounding has been shown to induce stomatal closure by activating COI1-dependent guard cell JA signaling, which in turn activates ABA biosynthesis and/or signaling [29]. Furthermore, studies using mutants deficient in ABA signaling showed that g_S_ responses induced by local stimuli were suppressed in these mutants [7,9,29,74]. Taken together, these data suggest that JA and ABA signaling pathways converge to induce decreases in g_S_, which mediates systemic photosynthetic responses to local stimuli.

**Figure 5:**
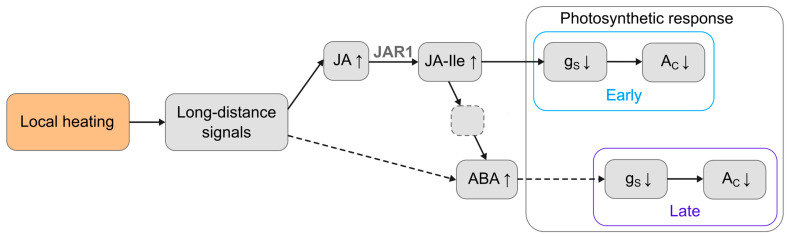
Working model for systemic photosynthetic responses to local heating mediated by the interaction between jasmonoyl-isoleucine (JA-Ile) and abscisic acid (ABA). Local heating induces the propagation of long-distance signals to systemic tissues. Long-distance signals trigger jasmonate biosynthesis and may also promote ABA production in systemic tissues. Rapid systemic increases in JA-Ile levels mediated by JASMONATE RESISTANT 1 (JAR1) activity contribute to ABA accumulation. The pathway by which JA-Ile regulates ABA accumulation is currently not clear. JA-Ile and ABA together induce a decrease in stomatal conductance (g_S_), which leads to a reduction in carbon assimilation (A_C_). While JA-Ile mediates the early stages, ABA contributes to late stages of the systemic photosynthetic response. Solid arrows indicate interactions based on the results of our study, and dashed arrows indicate hypothetical relationships that require further investigation.

The findings of the present study and published evidence support a model in which JA-Ile contributes to early stages, while ABA acts in late stages of systemic photosynthetic responses. Based on the results presented here and considering published data, the following working model of systemic photosynthetic responses mediated by a JA-Ile- and ABA-induced decrease in g_S_ is proposed (Figure 5).

## 4. Materials and Methods

### 4.1. Plant Material and Growth Conditions

All experiments were performed using *Arabidopsis thaliana* plants. The seeds of the *jar1-11* mutant in the Col-0 background and *35S:LOX2* transgenic line in the Col-6 (*gl1*) background were provided by the Nottingham *Arabidopsis* Stock Centre (NASC). For cold stratification, seeds were kept at 4 °C in darkness for 3 days. Plants were soil-grown under controlled environmental conditions in a Binder KBW 720 growth chamber (Binder GmbH, Tuttlingen, Germany) during 10 h of light (50 μmol m^−2^ s^−1^) at 23 °C and 14 h of dark at 21 °C with 65–70% humidity. For all measurements, 7-week-old plants were used.

Leaves of each plant were numbered sequentially from the oldest to the youngest according to a previously described method [17,55]. After numbering, leaf 13 was chosen as the stimulated leaf. Further, leaf 13 was designated as leaf *n*, and the younger leaves were designated as *n* + 2, *n* + 3, and *n* + 5, respectively, according to their numbering.

Measurements were performed locally in the stimulated leaf *n* and systemically in intact leaves *n* + 2, *n* + 3, and *n* + 5 (Figure 1f and Figure S5), which differ in their vascular connections with the local leaf *n*. Leaf *n* + 5 has direct vascular connections by parastichies to leaf *n*, and leaf *n* + 3 has contact parastichies to leaf *n*, but leaf *n* + 2 has a less direct connection to leaf *n* [55].

### 4.2. Local Stimulation and Surface Electrical Potential Recordings

Prior to the experiments, plants were transferred from the growth chamber to the recording room and allowed to acclimate for at least 1 h at approximately 23 °C. Local heating was applied to 40–50% of the leaf *n* using a water-filled cuvette. Water temperature during heating was monitored with an ATE-9380 temperature data logger (Aktakom, Moscow, Russia) and reached 60 °C for 6–7 min. A single local stimulation experiment was conducted for each individual plant.

To control VP propagation induced by local stimulation, monitoring of surface electrical potentials was used. Changes in electrical activity were monitored by Ag^+^/AgCl macroelectrodes EVL-1M3 (Gomel Plant of Measuring Devices, Gomel, Belarus) connected to a high-impedance amplifier IPL-113 (Semico, Novosibirsk, Russia) and a personal computer. Four measuring macroelectrodes were placed at the base of leaf blades (Figure 1f and Figure S5); the reference electrode was inserted into the soil.

### 4.3. Quantification of Phytohormones

Phytohormone concentrations, including SA, ABA, OPDA, JA and JA-Ile, were determined in the local leaf *n* and in systemic leaves *n* + 5, *n* + 3 and *n* + 2 (Figure 1f and Figure S5), with one leaf representing each sample. Leaves were collected at rest (0 min) and at defined time points (15, 30, 60 and 120 min) after VP propagation. Samples were immediately frozen in liquid nitrogen, then weighed, and ground into fine powder. Extraction of powdered leaf tissue and subsequent quantification of phytohormones were carried out according to the previously described protocols [24,43]. The triple quadrupole mass spectrometer LCMS-8040 (Shimadzu, Kyoto, Japan) was used to quantify levels of phytohormones. The VP propagation was monitored simultaneously with the analysis of phytohormones (Figure 1f and Figure S5). The same plants were used as a single experimental set for VP recording and phytohormone sampling.

### 4.4. Measurements of Gas Exchange and Chlorophyll Fluorescence

An infrared gas analyzer GFS-3000 (Heinz Walz GmbH, Effeltrich, Germany) equipped with a modulated light fluorometer (LED-ARRAY/PAM-Module 3055-FL) was used to measure dynamics of CO_2_ assimilation (A, μmol m^−2^ s^−1^), stomatal conductance (g_S_, mmol m^−2^ s^−1^) and chlorophyll fluorescence parameters such as the effective quantum yield of photochemical reactions of photosystem II (Φ_PSII_) and non-photochemical fluorescence quenching (NPQ), which was automatically calculated by GFS-Win software (v. 3.82) (Heinz Walz GmbH, Germany). The CO_2_ concentration in the measuring cuvette was 360 ppm, at 23 °C and 70% relative humidity. Red actinic light (175 µmol m^−2^ s^−1^, with 10% blue light) was used in the experiments. Illumination with a photon flux density of 3750 µmol m^−2^ s^−1^ was applied for 600 ms saturation pulses with a 60 s interval between them.

Measurements were performed on the systemic leaf *n* + 5. Plants were dark-adapted for 20 min. Illumination was then applied, and both gas exchange and chlorophyll fluorescence parameters were measured simultaneously for 75 min before local stimulation and for 90 min after stimulation. The sampling intervals were 1 s for gas exchange measurements and 60 s for chlorophyll fluorescence recordings.

### 4.5. Statistical Analysis

Each dataset was obtained from at least three independent biological replicates, which each replicate derived from a separate plant. In the case of phytohormone measurements, every biological replicate included three technical replicates. To obtain hormonal data, one leaf from one plant always constituted one biological replicate. Data processing was performed using MS Excel (Microsoft Corporation, Redmond, WA, USA). Statistical significance was evaluated using one-way ANOVA analysis followed by a Dunnett’s test and Student’s *t* test implemented in the GraphPad Prism software (v. 6.01, GraphPad Software Inc., San Diego, CA, USA). All comparisons between transgenic lines and background controls were performed using a pairwise Student’s *t* test. Comparisons within a time point were treated as planned comparisons, so no correction for multiple comparisons was applied. The details of the statistical analysis are described in the figure legends, including the number of biological repetitions and the statistical test.

## 5. Conclusions

This work enhances understanding of the hormone-related mechanisms regulating short-term systemic changes in photosynthetic activity induced by a long-distance signal, namely VP. The results of this study showed that local heating-induced systemic photosynthetic suppression was closely associated with reduced g_S_. In turn, the decrease in g_s_ was substantially mediated by JA-Ile, which also contributed to the production of ABA. The findings support a model in which JA-Ile and ABA collaborate to modulate systemic photosynthetic responses to local stimuli. Further research is needed to elucidate the pathway by which JA-Ile regulates ABA biosynthesis, as well as the exact mechanisms by which ABA and JA-Ile contribute to a decrease in g_S_.

## Data Availability

The original contributions presented in this study are included in the article/Supplementary Materials. Further inquiries can be directed to the corresponding author.

## Supplementary Materials

The following supporting information can be downloaded at: https://www.mdpi.com/article/10.3390/plants15111732/s1, Figure S1. Spatiotemporal dynamics of 12-oxophytodienoic acid (OPDA) (a), jasmonic acid (JA) (b), jasmonoyl-isoleucine (JA-Ile) (c), abscisic acid (ABA) (d) and salicylic acid (SA) (e) induced by heating of the leaf *n* in wild-type *Arabidopsis* plants (Col-0). Figure S2. Representative recordings of variation potential induced by local heating in wild-type *Arabidopsis* plants. Figure S3. Spatiotemporal dynamics of 12-oxophytodienoic acid (OPDA) (a), abscisic acid (ABA) (b), and salicylic acid (SA) (c) induced by heating of the leaf *n* in *35S:LOX2* *Arabidopsis* plants. Figure S4. Spatiotemporal dynamics of 12-oxophytodienoic acid (OPDA) (a), abscisic acid (ABA) (b), and salicylic acid (SA) (c) induced by heating of the leaf *n* in *jar1-11 Arabidopsis* plants. Figure S5. Experimental design for monitoring of surface electrical potentials, quantification of phytohormones, and monitoring of photosynthetic activity and stomatal conductance in *Arabidopsis* leaves.

## Abbreviations

The following abbreviations are used in this manuscript:

DAMPs	Damage-associated molecular patterns
ROS	Reactive oxygen species
VP	Variation potential
JA	Jasmonic acid
JA-Ile	(+)-7-*iso*-jasmonoyl-L-isoleucine
ABA	Abscisic acid
SA	Salicylic acid
OPDA	12-oxophytodienoic acid
LOX2	13-lipoxygenase 2
WT	Wild-type
JAR1	JASMONATE RESISTANT 1
Φ_PSII_	Effective quantum yield of photochemical reactions of photosystem II
NPQ	Non-photochemical fluorescence quenching
A	CO_2_ assimilation
g_S_	Stomatal conductance
GLR	GLUTAMATE RECEPTOR-LIKE
ABA-GE	ABA glucosyl ester
NCED2	9-CIS-EPOXYCAROTENOID DIOXYGENASE 2
ABI5	ABA INSENSITIVE 5
BGLU18	β-GLUCOSIDASE 18
COI1	CORONATINE INSENSITIVE 1
