# Supplementary material for: Jasmonoyl-L-Isoleucine Induces Systemic Photosynthetic Responses to Local Heat Stress by Contributing to Abscisic Acid Accumulation
Source: Plants (Basel). 2026 Jun 3;15(11):1732. doi: 10.3390/plants15111732 (PMC13259213; doi:10.3390/plants15111732)
Supplement: Supplementary file 1 [file plants-15-01732-s001.zip › plants-4306335-supplementary.pdf]

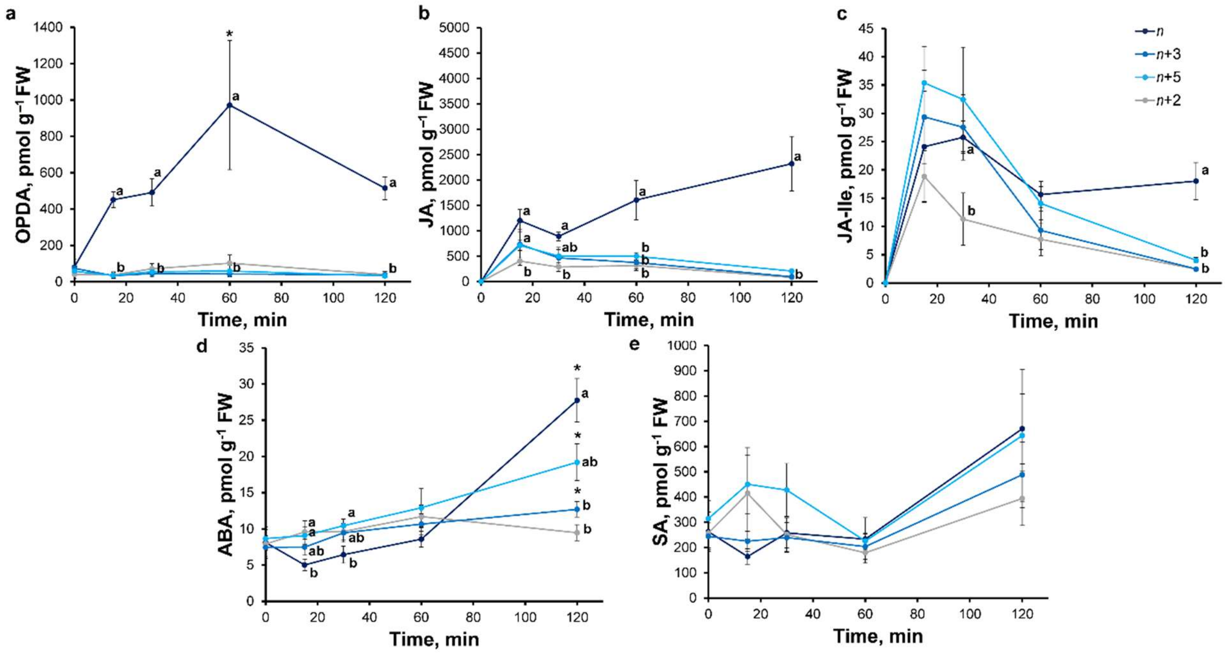

Figure S1. Spatiotemporal dynamics of 12-oxophytodienoic acid (OPDA) (**a**), jasmonic acid (JA) (**b**), jasmonoyl-isoleucine (JA-Ile) (**c**), abscisic acid (ABA) (**d**) and salicylic acid (SA) (**e**) induced by heating of the leaf *n* in wild-type *Arabidopsis* plants (Col-0). The variation potential (VP) propagation was monitored simultaneously with the analysis of hormones. The moment of VP propagation corresponds to the time point "0 min". The same plants were used as a single experimental set for VP recording and phytohormone sampling. Data are represented as Mean ± SEM (n = 3 to 6). Asterisks indicate data significantly different from unstimulated plants (0 min) according to one-way ANOVA ($p < 0.05$). Different letters indicate statistically significant differences between leaves within a time point according to Student's t test ($p < 0.05$).

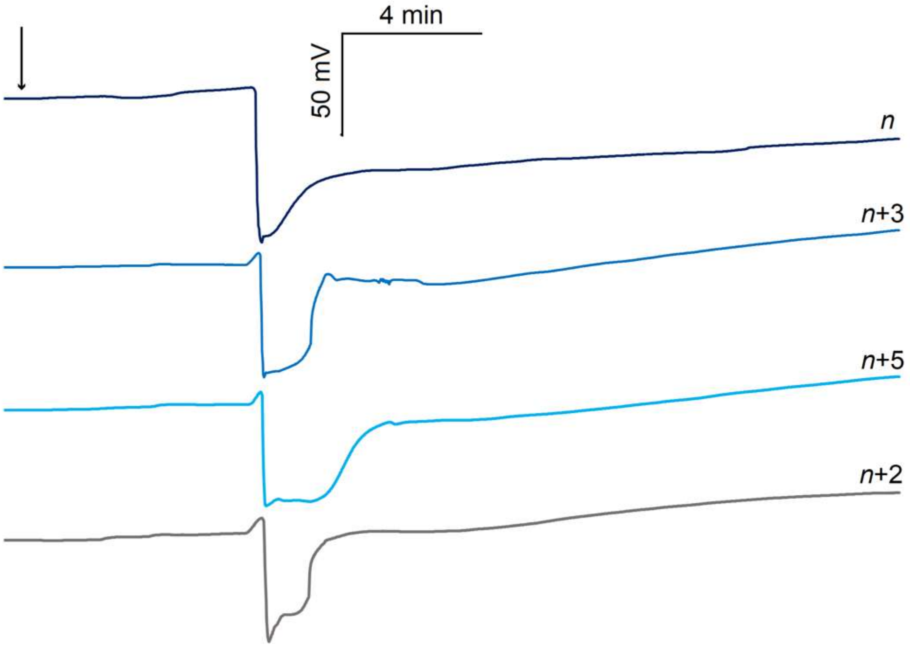

Figure S2. Representative recordings of variation potential induced by local heating in wild-type *Arabidopsis* plants. The arrow indicates the moment of beginning of heating of the leaf *n*.

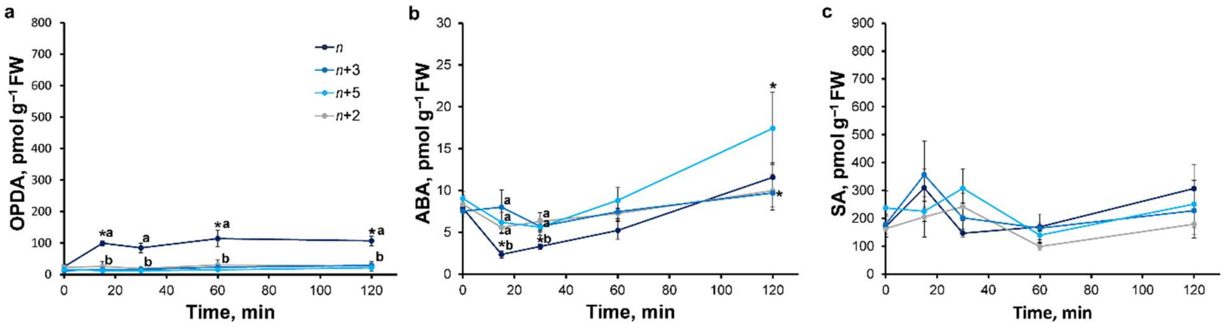

Figure S3. Spatiotemporal dynamics of 12-oxophytodienoic acid (OPDA) (**a**), abscisic acid (ABA) (**b**), and salicylic acid (SA) (**c**) induced by heating of the leaf *n* in *35S:LOX2 Arabidopsis* plants. The variation potential (VP) propagation was monitored simultaneously with the analysis of hormones. The moment of VP generation corresponds to the time point "0 min". The same plants were used as a single experimental set for VP recording and phytohormone sampling. Data are represented as Mean ± SEM (n = 3 to 6). Asterisks indicate data significantly different from unstimulated plants (0 min) according to one-way ANOVA ($p < 0.05$). Different letters indicate statistically significant differences between leaves within a time point according to Student's *t* test ($p < 0.05$).

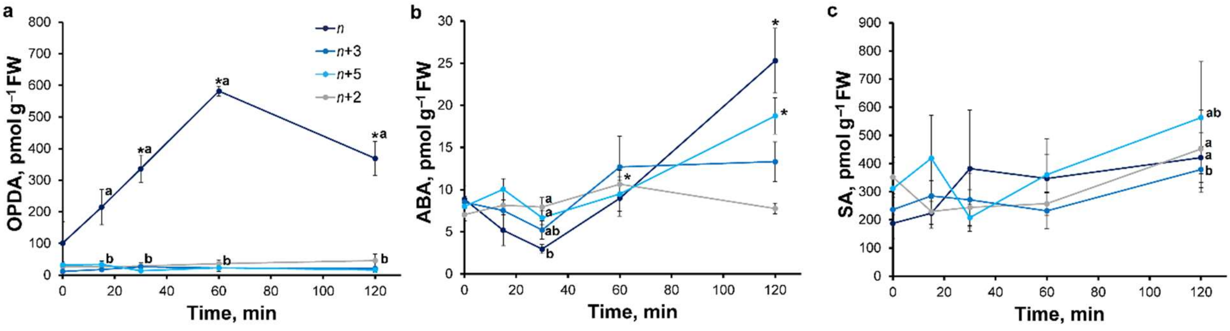

Figure S4. Spatiotemporal dynamics of 12-oxophytodienoic acid (OPDA) (**a**), abscisic acid (ABA) (**b**), and salicylic acid (SA) (**c**) induced by heating of the leaf *n* in *jar1-11 Arabidopsis* plants. The variation potential (VP) propagation was monitored simultaneously with the analysis of hormones. The moment of VP generation corresponds to the time point "0 min". The same plants were used as a single experimental set for VP recording and phytohormone sampling. Data are represented as Mean ± SEM (n = 3 to 6). Asterisks indicate data significantly different from unstimulated plants (0 min) according to one-way ANOVA ($p < 0.05$). Different letters indicate statistically significant differences between leaves within a time point according to Student's *t* test ($p < 0.05$).

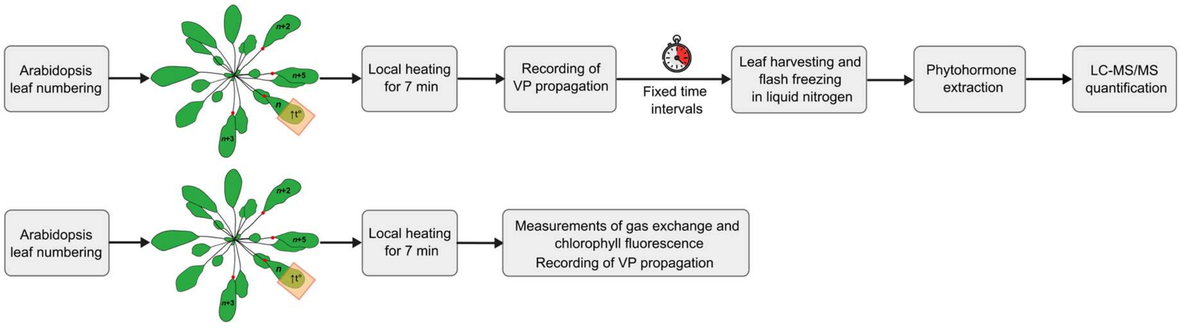

Figure S5. Experimental design for monitoring of surface electrical potentials, quantification of phytohormones, and monitoring of photosynthetic activity and stomatal conductance in *Arabidopsis* leaves. Leaves of each plant were numbered sequentially from the oldest to the youngest. Red circles indicate surface electrodes. Phytohormone levels were analyzed using the same biological material used from analysis of variation potential (VP). To obtain hormonal data, one leaf from one plant always constituted one biological replicate.
